# The Early and Long-Term Outcomes of Total Arch Replacement Using the Frozen Elephant Trunk (FET) Technique for Chronic Aortic Aneurysms

**DOI:** 10.7759/cureus.78664

**Published:** 2025-02-07

**Authors:** Taro Kuroda, Koji Tsutsumi, Osamu Ishida

**Affiliations:** 1 Department of Cardiovascular Surgery, National Defense Medical College, Tokorozawa, JPN

**Keywords:** aortic surgery, chronic aortic aneurysms, frozen elephant trunk technique, surgical technique, total arch replacement

## Abstract

Background: The favorable long-term outcomes have been reported for total arch replacement using the frozen elephant trunk technique for acute aortic dissection. However, the data on chronic degenerative aortic aneurysms treated with this technique are limited compared with those for acute aortic dissection, and the long-term outcomes are not well understood. Here, we report early and long-term outcomes of total arch replacement using the frozen elephant trunk technique in patients with chronic aortic aneurysms.

Methods: We included 37 patients with chronic degenerative aortic aneurysms who underwent total arch replacement using the frozen elephant trunk technique between January 2015 and December 2022 at the National Defense Medical College Hospital, Tokorozawa, Japan. The primary endpoints were the 30-day and five-year survival rates. The secondary endpoints were major adverse cardiac and cerebrovascular events and acute renal failure.

Results: The 30-day mortality rate was 13.5%, and the factors influencing this rate were postoperative cerebral infarction, acute renal failure, and prolonged ventilation. The factors influencing major adverse cardiac or cerebrovascular events and acute renal failure included a history of preoperative stroke. The five-year survival rate was 61.1%.

Conclusion: Total arch replacement using the frozen elephant trunk technique for degenerative aortic aneurysms at our institution yielded satisfactory early and long-term surgical outcomes.

## Introduction

Total arch replacement is a standard procedure in treating aortic arch pathologies. Recent developments in surgical procedures and cerebral protection techniques have led to excellent surgical outcomes; however, it remains a challenging procedure for pathologies extending into the proximal descending aorta [1,2]. The frozen elephant trunk (FET) technique allows for the replacement of the arch and descending thoracic aorta in a single operation, which can reduce the patient’s overall risk and recovery time [3-6]. In Japan, the use of the J Graft Frozenix (Tokyo, Japan: Japan LifeLine) device for the FET technique was approved for insurance coverage in 2014; since then, use of the FET technique has spread rapidly [4]. Total arch replacement using the FET technique has been widely employed for the treatment of acute aortic dissection [7], with numerous favorable long-term results reported in recent years [3,5,6]. In comparison, however, there are relatively few reports on chronic aortic aneurysms treated with this technique, particularly with insufficient understanding of the long-term outcomes. We previously reported the surgical outcomes of total arch replacement using the FET technique (specifically, the J Graft Frozenix) for chronic degenerative distal aortic arch aneurysms at two institutions, including our institute, in which we demonstrated favorable mid-term results [8].

Therefore, through this study, we report the early and long-term outcomes of total arch replacement using the FET technique for chronic degenerative aortic aneurysms at our institute.

## Materials and methods

Patients and study design

This was a single-center observational study. Patients who underwent emergency surgery were excluded. Patients with atherosclerotic aortic arch aneurysms originating in the ascending aorta or aortic arch and extending to the proximal descending aorta at the T7 (thoracic 7) level were included. Patients with descending aortic aneurysms beyond T8 (thoracic 8) were excluded. A shaggy aorta was defined as follows: (i) thrombus measurement in non-aneurysmal aortic segments <40 mm, (ii) atheroma thickness ≥5 mm, and (iii) irregular atheroma surface showing finger-like projections. If the patient had all three findings, the diagnosis of a shaggy aorta was confirmed [9].

Between January 2015 and December 2022, at the National Defense Medical College Hospital in Tokorozawa, Japan, 37 patients received total arch replacement using the FET technique for chronic degenerative aortic aneurysms. All patients underwent a preoperative computed tomography (CT) scan with contrast. The patient records were retrospectively reviewed. The Institutional Review Board of the National Defense Medical College approved this study (#4294; approved on October 6, 2020) and waived the requirement for individual consent.

Surgical techniques

The surgical techniques are similar to those that we have reported in the past [8]. Preoperative cerebrospinal fluid drainage was not routinely performed. Cardiopulmonary bypass was initiated by arterial cannulation of the ascending aorta and the right subclavian artery and bicaval venous cannulation. Myocardial protection was achieved with intermittent antegrade or retrograde cold blood cardioplegic solution depending on the condition of the patient’s ascending aorta. Near-infrared spectroscopy was used for cerebral monitoring. When the rectal temperature reached 25°C, circulatory arrest was started. Cooling was maintained at rectal temperatures of 20°-25°C. The aortic arch is usually transected circumferentially, just distal to the origin of the left common carotid artery, followed by cerebral protection using antegrade selective cerebral perfusion (ASCP). The ASCP was maintained through cannulation of the brachiocephalic and left common carotid arteries, with additional cannulation of the left subclavian artery in cases of the dominant left vertebral artery. The Frozenix J graft was deployed antegrade to the descending aorta, and its size and length were selected as previously described [8]. After the graft portion of the J Graft Frozenix was trimmed and incorporated into the native aortic stump wall, they were anastomosed together with the proximal four-branched prosthesis. The left subclavian artery was ligated at its origin and reconstructed by the side branch of the proximal prosthesis in an end-to-end or end-to-side fashion, depending on the condition of the patient’s left subclavian artery. Finally, proximal anastomosis was performed.

Endpoints and follow-ups

This study assessed the following two primary endpoints: the 30-day mortality and five-year survival. The secondary endpoints were risk factors for major adverse cardiac or cerebrovascular events (MACCE) and acute kidney injury (AKI). MACCE were defined as either the 30-day mortality or major cardiovascular or cerebrovascular events. AKI was defined as meeting either of the following two criteria and not being placed on chronic dialysis: (1) serum creatinine level increased more than twice from the baseline or (2) urine output <0.5 mL/kg/h for ≥12 h [10].

Statistical analysis

Data were collected and analyzed retrospectively. Categorical and continuous variables are summarized as percentages and medians with interquartile ranges, respectively. Statistical analysis was performed using Fisher's exact probability test, and a p-value less than or equal to 0.05 was considered statistically significant. All statistical analyses were performed using EZR version 1.36 (Saitama, Japan: Saitama Medical Center, Jichi Medical University), which is a graphical user interface for R (Vienna, Austria: The R Foundation for Statistical Computing) [11]. More precisely, it is a modified version of the R commander designed to add statistical functions frequently used in biostatistics.

## Results

Preoperative data

Patient demographics and backgrounds are summarized in Table 1. The median age was 77 years (interquartile range: 72-80 years), and 33 patients (89.2%) were male. Two patients had a history of open-heart surgery. The median of the calculated EuroSCORE II was 5.47%. Shaggy aorta was observed in as many as 26 cases (70.2%). ASCP was maintained through the brachiocephalic and left carotid arteries in 24 cases (64.9%), whereas ASCP through the left subclavian artery was performed in 13 cases (35.1%). Concomitant retrograde cerebral perfusion was performed before ASCP in four patients. Usually, the aortic arch is cut between left common and left subclavian arteries, with distal anastomosis performed at the level of zone 2 in 28 patients (75.7%). Zones 1 and 3 anastomoses were chosen depending on the condition of the aortic arch (one case {2.7%} and eight cases {21.6%}, respectively). The J Graft Frozenix was successfully implanted in 37 patients without any complications. The median diameter of the implanted grafts was 30 mm and the median length was 90 mm. Concurrent surgical procedures were performed in three cases (coronary artery bypass grafting in one case and aortic valve replacement in two cases).

**Table 1:** Preoperative characteristics of the patients included in this study. Continuous data are reported as median and IQR. Categorical data are reported as number (%). COPD: chronic obstructive pulmonary disease; CKD: chronic kidney disease; IQR: interquartile range

Variable	Value (n=37)
Age (years)	77 (72, 80)
Sex (male) (%)	33 (89.2)
BMI	22.9 (20.9, 24.6)
Hypertension (%)	32 (86.5)
Diabetes (%)	9 (24.3)
Dyslipidemia (%)	13 (35.1)
Smoking (%)	30 (81.1)
COPD (%)	19 (51.4)
Peripheral vascular disease (%)	14 (37.8)
Abdominal aortic aneurysms (%)	11 (29.7)
CKD stage II, III (%)	19 (51.4)
Hemodialysis (%)	1 (2.7)
Cerebral infarction (%)	11 (29.7)
Heart failure (%)	3 (8.1)
Reoperation (%)	2 (5.4)
Aneurysmal diameter (mm)	55 (48, 60)
Fusiform (%)	24 (64.9)
Saccular type (%)	13 (35.1)
Shaggy aorta	26 (70.2)
EuroScore II	5.47 (4.42, 11.5)

Operative data

The operative parameters and findings are shown in Table 2. The median operative, cardiopulmonary bypass (CPB), aortic clamp, and hypothermic circulatory arrest (HCA) times were 498, 296, 209, and 84 minutes, respectively.

**Table 2:** Surgical procedures performed and the surgical data for the patients included in this study. Continuous data are reported as median and IQR. Categorical data are reported as number (%). CPB: cardiopulmonary bypass; BCA: brachiocephalic artery; LCA: left carotid artery; LSCA: left subclavian artery; IQR: interquartile range; Retro: retrograde

Variable		Value (n=37)
Surgical time (min)		498 (442, 538)
CPB time (min)		296 (265, 312)
Cross clamp time (min)		209 (186, 214)
Deep hypothermic arrest time (min)		84 (75, 96)
Selective cerebral perfusion time (min)		139 (126, 153)
Selective cerebral perfusion (%)	BCA+LCA	21 (56.8)
	BCA+LCA+retro	3 (7.7)
	BCA+LCA+LSCA	12 (32.4)
	BCA+LCA+LSCA+retro	1 (2.7)
Distal landing zone (%)	T 4	1 (2.9)
	T 5	6 (17.6)
	T 6	15 (44.1)
	T 7	10 (29.4)
	T 8	2 (5.9)
Open stent graft diameter (mm) (%)	25	2 (5.4)
	27	3 (8.1)
	29	9 (24.3)
	31	15 (40.5)
	33	3 (8.1)
	35	5 (13.5)
Open stent graft length (mm) (%)	60	6 (16.2)
	90	22 (59.5)
	120	9 (24.3)
Prosthetic graft diameter (mm) (%)	24	1 (2.7)
	26	3 (8.1)
	28	16 (43.2)
	30	17 (45.9)
Concurrent surgical procedure		3 (7.7)

ASCP was maintained through the brachiocephalic and left carotid arteries in 24 cases (64.9%), whereas ASCP through the left subclavian artery was performed in 13 cases (35.1%). Concomitant retrograde cerebral perfusion was performed before ASCP in four patients. Usually, aortic arch is cut between left common and left subclavian arteries, with distal anastomosis performed at the level of zone 2 in 28 patients (75.7%). Zones 1 and 3 anastomoses were chosen depending on the condition of the aortic arch (one case {2.7%} and eight cases {21.6%}, respectively). The J Graft Frozenix was successfully implanted in 37 patients without any complications. The median diameter of the implanted grafts was 30 mm and the median length was 90 mm. Concurrent surgical procedures were performed in three cases (coronary artery bypass grafting in one case and aortic valve replacement in two cases).

Postoperative results

Postoperative results are shown in Table 3. The 30-day mortality rate was 13.5%, with stroke accounting for 80% of the deaths. Permanent stroke was observed in eight patients (21.6%), spinal cord injury occurred in one patient (2.7%), and AKI was observed in 12 patients (32.4%).

**Table 3:** Postoperative results for the patients included in the study. Continuous data are reported as median and IQR. Categorical data are reported as number (%). AKI: acute kidney injury; ARDS: acute respiratory distress syndrome; IQR: interquartile range

Variable		Value (n=37)
Prolonged ventilation (>96 hours, %)		7 (18.9)
Pneumonia (%)		2 (5.4)
AKI (%)		12 (32.4)
Hemodialysis required (%)		3 (8.1)
Mediastinitis (%)		0 (0)
Permanent stroke (%)		8 (21.6)
Spinal cord injury (%)		1 (2.7)
Recurrent nerve palsy (%)		4 (10.8)
Endoleakage (%)		1 (3.6)
Re-exploration (%)		0 (0)
ICU stay (day)		3 (3, 5)
30-day mortality (%)		5 (13.5)
Cause of death	Stroke	4
	ARDS	1

The postoperative risk factors related to 30-day mortality, along with their p-values, odds ratios, and 95% confidence intervals, are presented in Table 4. Among these risk factors, permanent stroke and prolonged ventilation were strongly associated with 30-day mortality. AKI was also significantly associated with 30-day mortality. As for the secondary end-point analysis, preoperative stroke history showed a significant association with the postoperative MACCE and AKI occurrence (odds ratio=6.8 {1.21-51.6}, p=0.03).

**Table 4:** Postoperative risk factors related to 30-day mortality. CI: confidence interval; AKI: acute kidney injury

Variables	Odds ratio	95% CI	p-Value
Permanent stroke	24.0	1.84-1409	0.005
Recurrent nerve palsy	2.3	0.04-39.48	0.45
AKI	11.1	0.93-612	0.03
Prolonged ventilation (>96 hours)	31.8	2.31-1932	0.0002
Pneumonia	7.0	0.08-623	0.26

**Figure 1:**
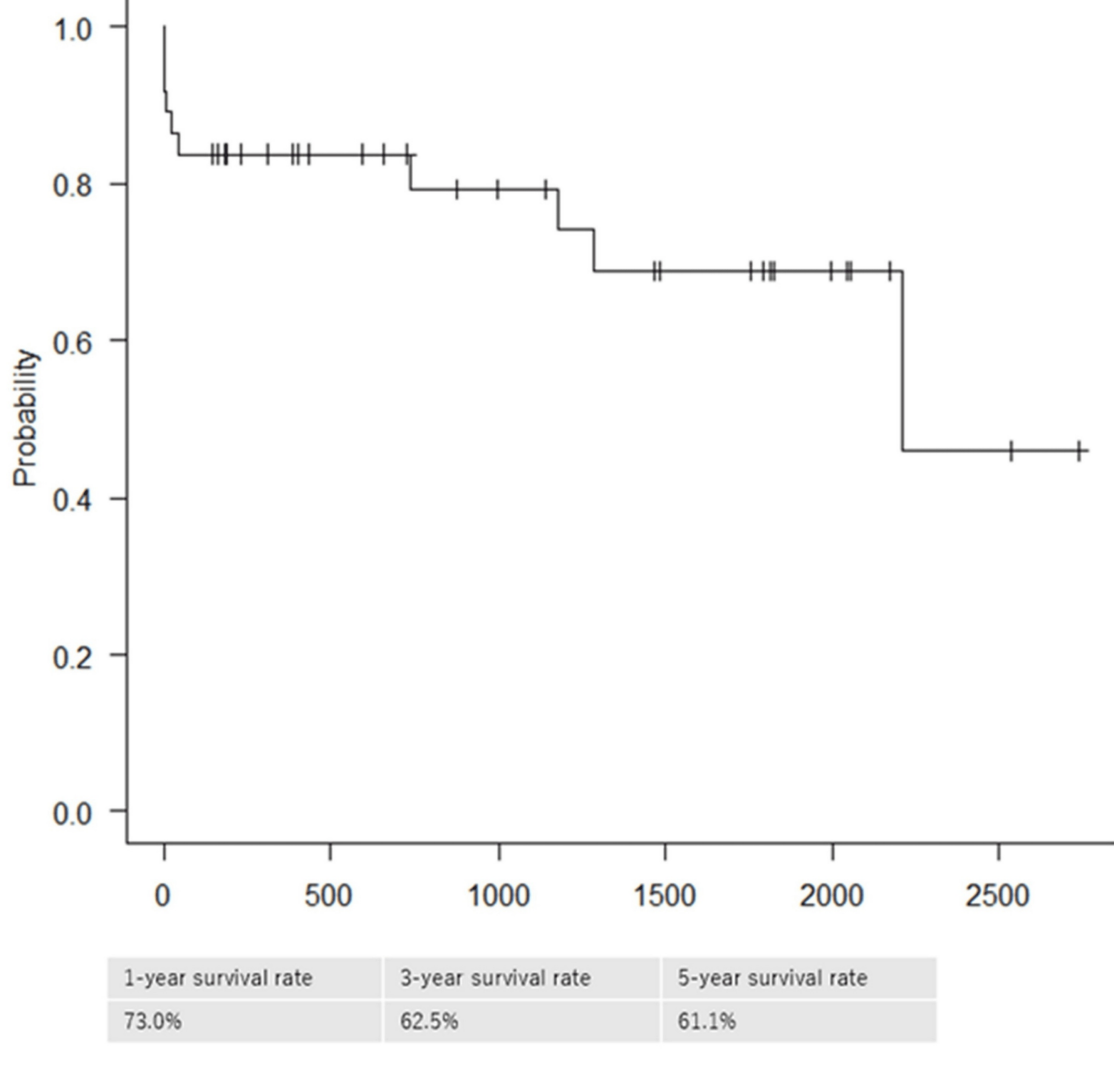
Kaplan-Meier survival curve for all patients undergoing surgery (n=37).

The Kaplan-Meier curve of the patients during the observation period is presented in Figure 1. Thirty-seven patients were traceable. The one-year, three-year, and five-year survival rates were 73.0%, 62.5%, and 61.1%, respectively. There were no statistically significant factors that contributed to the five-year survival rate.

## Discussion

Recently, in the aging population, the number of patients with extensive degenerative aortic disease involving the distal aortic arch has increased [4]. Due to the complex surgical strategy for aortic arch repair and the limited access to aneurysms in the descending aortic arch via a median sternotomy, distal aortic arch aneurysms remain a significant challenge for cardiovascular surgeons. In 1996, Kato et al. reported a combination of traditional total arch replacement and an endovascular technique known as "open stent grafting" [12]. In 2003, Karck et al. first described the FET technique for descending aortic aneurysms or chronic aortic dissection using total arch replacement and stent graft insertion into the descending aorta [13]. Since then, the indications for FET have expanded considerably. This new approach has become an alternative strategy for extensive aortic disease, as it allows the complete surgical treatment of combined aortic lesions in a single operation via median sternotomy. Previous studies indicated that this technique is feasible for long-term outcomes in acute aortic dissection [3,6]. However, limited information is available on the long-term outcomes of total arch replacement using FET for chronic aortic arch lesions. We have reported the surgical outcomes of total arch replacement using the FET technique (J Graft Frozenix) for chronic degenerative distal aortic arch aneurysms at two institutions, including our institution [8]. In that report, we included 59 cases and demonstrated favorable mid-term results, revealing an 84.8% one-year survival rate and a 79.4% three-year survival rate at a median follow-up of 1.8 years. As a follow-up to these results, the current report analyzed patients at our institute with a focus on long-term outcomes.

According to the collected literature reports, an early mortality rate of total arch replacement using the FET technique for distal chronic aortic arch lesions was 2.5-19.2% [14-18]. The 30-day mortality rate in this study cohort was 13.5%, which is comparable to the results of previous reports. Permanent stroke accounted for 80% of patient mortality and was also significantly associated with 30-day mortality. When the distribution of cerebral infarcts in this cohort was examined separately, most infarcts occurred in the basilar artery region, suggesting that the embolus flowed from the left subclavian artery during FET deployments [8]. Therefore, we modified the procedure to ligate the left subclavian artery before the FET deployment, which greatly reduced the occurrence of cerebral infarcts. Manipulation of the arch is critical in traditional total arch replacement with respect to the occurrence of stroke [1]. However, it needs to be carefully addressed in FET, especially in patients with a shaggy aorta, as in this cohort.

AKI was significantly associated with 30-day mortality and the incidence of AKI was relatively high compared to previous reports [5]. The etiology of AKI after cardiac surgery is multifactorial [19], whereas longer CPB and cross-clamp times are associated with the occurrence of AKI in a dose-response manner [20]. Aortic surgery is associated with a higher incidence of AKI [21] and is commonly associated with increased morbidity and mortality after cardiac surgery [19]. Several reports of patients who underwent total arch replacement using the FET technique have documented that AKI is a risk factor for postoperative mortality [22,23].

In terms of factors influencing the combined risk of MACCE and AKI, preoperative stroke history was the only factor identified. Since preoperative stroke history is a well-known risk factor for postoperative stroke after total arch replacement, we consider this a reasonable finding [24,25]. As for the other factors that were not included, this may be due to the insufficient extraction power of this study.

The five-year survival rate was found to be at 61.1%. A systematic review of the long-term results showed a five-year survival rate of 79.1% for total arch replacement using the FET technique in patients with chronic aortic aneurysms or chronic aortic dissection, with a median age of 55 years [5]. Tokunaga et al. reported a five-year survival rate of 65.4% using the same device as this study, with a mean age of 74.3 years [16]. Considering that the median age of our cohort was 77 years, we believe that our five-year survival rate was acceptable.

There were some limitations to this study. Firstly, it was a retrospective study from a single institution and the sample size was small. Thus, the reproducibility of the results obtained may differ and may not apply to the entire population of patients with chronic degenerative distal aortic arch aneurysms. Secondly, to conclude on the long-term results of total arch replacement using the FET technique, the follow-up period was relatively short. However, few studies have addressed the repair of distal aortic arch aneurysms with total arch replacement using the FET technique, and our findings provide support for it as an effective therapeutic option. Further studies with a larger cohort and longer follow-up are therefore needed.

## Conclusions

Total arch replacement using the FET technique for degenerative aortic aneurysms at our institution has shown acceptable 30-day mortality and five-year survival rates. The risk factors for 30-day mortality were permanent stroke, prolonged mechanical ventilation, and AKI. The risk factor for MACCE and AKI was preoperative stroke history. Total arch replacement using the FET technique for degenerative aortic aneurysms at our institution has shown satisfactory five-year survival rates.
